# Assessment of Serum Concentrations of Ghrelin, Obestatin, Omentin-1, and Apelin in Children with Type 1 Diabetes

**DOI:** 10.1155/2016/8379294

**Published:** 2016-01-19

**Authors:** Agnieszka Polkowska, Izabela Szczepaniak, Artur Bossowski

**Affiliations:** ^1^Department of Pediatrics, Endocrinology, Diabetology with Subdivision of Cardiology, Medical University of Białystok, Ulica Kilińskiego 1, 15-089 Białystok, Poland; ^2^Department of Pediatrics, Endocrinology, Diabetology with Subdivision of Cardiology, Children's University Hospital in Białystok, Ulica Waszyngtona 17, 15-274 Białystok, Poland

## Abstract

The increasing knowledge on the functions of gastric peptides and adipokines in the body allows the assumption of their major role linking the process of food intake, nutritional status, and body growth, largely through the regulation of glucose metabolism and insulin resistance. The aim of the study was the assessment of serum levels of selected gastric peptides and adipocytokines in children with type 1 diabetes, with respect to the disease duration. The study involved 80 children aged 4–18 years (M/F -37/43). Children with type 1 diabetes (*n* = 46) were compared to the control group (*n* = 34). The study group was divided into 4 subgroups: (I) patients with newly diagnosed type 1 diabetes, after an episode of ketoacidosis (*n* = 10), (II) patients with type 1 diabetes of duration no longer than 5 years (*n* = 9), (III) patients with 5 to 10 years of DT1 (*n* = 20), and (IV) patients with type 1 diabetes of duration longer than 10 years (*n* = 7). The concentrations of gastric peptide and adipocytokines across all subgroups were lower than in the control group. The differences were statistically significant (*p* < 0.0001), which may be of importance in the development of the disease complications.

## 1. Introduction

The increasing knowledge on the functions of intestinal peptides and adipokines in the body allows the assumption of their major role linking the process of food intake, nutritional status, and body growth, largely through the regulation of glucose metabolism and insulin resistance. The alimentary tract and human adipose tissue constitute an important part of the endocrine system, producing the greatest amounts of regulatory peptides. Food intake control is substantially regulated by the hypothalamic neurons that receive various signals from the so-called circuit-adipose tissue, stomach, intestines, thyroid, or pancreas [1, 2].

One of the adipokines is omentin, a protein produced mainly in visceral adipose tissue. Its function is most likely to increase insulin sensitivity and stimulate glucose metabolism, and this effect can be both local and systemic. In vitro studies have demonstrated that omentin increases insulin sensitivity by stimulating insulin-dependent glucose uptake in both subcutaneous and visceral adipocytes [3]. Since omentin is produced by visceral and not subcutaneous adipose tissue, its function can be compared to that of visfatin [4]. Visfatin also works synergistically with insulin and increases the insulin-dependent glucose uptake. However, unlike visfatin, omentin does not affect the basic glucose transport. The existence of insulin-independent effects of omentin is indirectly manifested by Akt/PKB (Protein Kinase B) phosphorylation, which occurs under the influence of this mediator in both presence and absence of insulin. Omentin is also a useful biomarker for endothelial function, since the level of omentin circulating in blood is an independent factor responsible for the presence of atherosclerotic plaques in patients with type 2 diabetes [5]. In the circulation only omentine-1 is detected. The highest concentration of this isoform has been demonstrated in visceral adipose tissue. On the other hand, omentin-2 is secreted mainly to the intestine. This explains why omentin-2 is not detected in serum [6].

Another adipokine is apelin, which has been shown to enhance sensitivity of cells to insulin and delay the development of metabolic disorders associated with obesity [7], to a large extent by strong inotropic and hypotensive effects [8].

Ghrelin and obestatin are hormones secreted by the digestive tract, mainly the parietal cells of the gastric fundus [9]. They have a common source, preproghrelin, and show opposite effects [10, 11], although some literature reports cast doubt on their antagonistic activity [12]. Ghrelin is called the hunger hormone; its expression and secretion are increased when energy balance is negative. It remains an independent predictor of insulin resistance [13]. In contrast, obestatin is a satiety hormone.

Most papers evaluating the relationship of gastrointestinal peptides and adipocytokines with glucose metabolism provide data obtained from studies conducted on animal models or adults with type 2 diabetes. There are only few reports assessing these relationships in young patients with type 1 diabetes.

*Aim of the Study*. The study objective was to evaluate the concentrations of selected gastric peptides and adipocytokines in blood serum of children with type 1 diabetes, taking into account the disease duration.

## 2. Materials and Methods

The study included 80 children aged 4–18 years (M/F -37/43), diabetic patients of Diabetology Outpatient Clinic and Department of Pediatrics, Endocrinology, Diabetology with Subdivision of Cardiology, Medical University of Bialystok. The group of 46 children with type 1 diabetes (diagnosed by ISPAD criteria) was compared to the control group of 34 healthy children with a negative history of inflammatory diseases, autoimmune diseases, or cancer. For the purpose of the study, the diabetic group was divided into 4 subgroups: (I) patients with newly diagnosed type 1 diabetes, after an episode of ketoacidosis (*n* = 10), (II) patients with type 1 diabetes of duration no longer than 5 years (*n* = 9), (III) patients with 5 to 10 years of DT1 (*n* = 20), and (IV) patients with type 1 diabetes of duration longer than 10 years (*n* = 7).

We analyzed such anthropometric parameters as age, height, and weight, laboratory tests (HbA1c, lipids, TSH, and antibodies anti-thyroid) using standard methods, as well as ghrelin, apelin, and omentin-1, and obestatin measured by ELISA. Statistical analysis was performed using Statistica 10.0. The nonparametric Mann-Whitney *U* test was used to compare the quantitative variables without normal distribution.

## 3. Results

**Figure 1:**
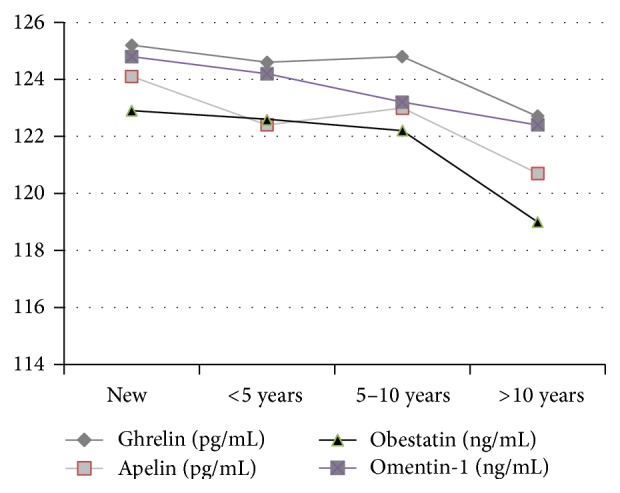
The concentration of ghrelin, obestatin, omentin-1, and apelin in the serum of children with type 1 diabetes, with respect to the disease duration.

In all the study subgroups, the levels of gastric peptides and adipocytokines were lower than in the control group. The differences between the respective subgroups and the control group were statistically significant (*p* < 0.0001) (Table 1). Moreover, we observed the lowest levels of all the regulatory peptides in the subgroup of children with the longest disease duration (Figure 1).

**Figure 2:**
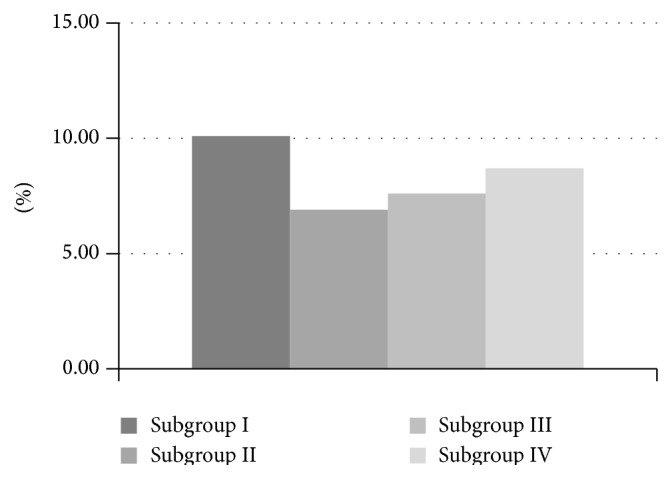
Mean HbA1c values in subgroups with type 1 diabetes. Statistical significance between subgroup I and subgroup II, *p* < 0.001. Statistical significance between subgroup I and subgroup III, *p* < 0.002. Lack of statistical significance between subgroup I and subgroup IV.

The glycated hemoglobin level was the highest in subgroup I (10.1%), due to the fact that these were recently diagnosed cases of type 1 diabetes. The mean HbA1c among children in the subgroups II and III were comparable (6.9% vs. 7.6%). Higher HbA1c (8.7%) was observed in the group of children with the longest disease duration and thus with the lowest level of the peptides tested (Figure 2).

The analysis of the anthropometric parameters revealed that the groups did not differ statistically significantly in terms of body height. Only the children with newly diagnosed diabetes were significantly younger (*p* = 0.029) and had a statistically significantly lower body weight (*p* = 0.028) as compared to the control group, whereas patients with the longest disease duration and the lowest levels of the substances tested had a higher BMI than healthy children (*p* = 0.027) (Table 2).

Moreover, we noted a statistically significant negative correlation between BMI and the levels of all the regulatory substances tested, between body weight and the levels of obestatin, ghrelin, and apelin in children with the longest disease duration and between BMI and the concentrations of obestatin and apelin in children with 5 to 10 years' lasting diabetes.

## 4. Discussion

Hormones secreted by the digestive tract and adipose tissue are important regulators of energy metabolism. Numerous studies concerning different fields of medicine are still discovering their novel properties. Hormones are involved in many biological processes which affect not only the function of the gastrointestinal tract but also endocrine and immune systems, the processes of growth and maturation. Accurate knowledge of the mechanisms of regulatory peptides and factors that influence their release may create new possibilities not only in the treatment of obesity and inflammatory diseases but also in the treatment of type 1 diabetes.

The results of the present analysis show a negative correlation of regulatory peptides with body mass and BMI. Similar correlations were observed in a study conducted by Tapan et al., showing significantly lower plasma apelin concentration in obese children as compared to children with normal weight [14]. Reports by Ziora et al. indicate a statistically significant negative correlation between plasma levels of ghrelin and BMI in girls with anorexia nervosa [15]. Similar relationships were confirmed by de Souza Batista et al. [6] and Zamrazilová et al. [16] among adults, where lower concentrations of omentin and obestatin were associated with higher BMI.

Our analysis indicates that the lower levels of regulatory substances are accompanied by a higher BMI and increased glycated hemoglobin. However, there are reports in which positive correlation was observed between apelin level and body mass index (BMI), HOMA-IR, triglycerides, glucose, and glycated hemoglobin (HbA1c) [17]. However, authors' opinions as to which of these factors exert the greatest effect on the level of apelin vary.

In the present analysis, a subgroup of patients with the longest duration of the disease had the lowest levels of the peptides tested. The available data indicate that apart from numerous known actions of the digestive hormones and adipocytokines, there are others that affect proliferation and survival of pancreatic isles cells [18], which may suggest a relationship between the level of the regulatory peptides and remission duration.

Insulin resistance affects the clinical course and metabolic control in patients with both type 2 and type 1 diabetes. In children with type 1 diabetes, insulin resistance is higher than in the group of their peers [19]. The available data emphasize that it is insulin resistance and not obesity that causes suppression of ghrelin secretion. However, there is no agreement whether lower level of ghrelin is the result or the cause of insulin resistance [13]. This may explain why the patients with the longest disease duration and high mean glycated hemoglobin showed the lowest concentrations of the regulatory peptides.

A significantly higher than normal risk of developing atherosclerosis in diabetic patients can be explained by a higher incidence of classical risk factors for developing cardiovascular diseases in these patients, adverse effects of diabetes on other risk factors, such as dyslipidemia and hypertension, and the direct effect of hyperglycemia or diabetes itself [20, 21]. Cardiovascular complications in the course of diabetes begin to develop in childhood [22, 23]. According to the guidelines of the American Heart Association, type 1 diabetes in children and adolescents can be regarded as the equivalent of ischemic heart disease. However, the accelerated development of atherosclerosis begins with the development of diabetes and has a close connection not only with traditional risk factors but also with metabolic control and as research reports, with blood levels of the regulatory peptides. Younger patients who have not yet developed ischemic heart disease show early structural changes, such as increased thickness of the inner and middle vascular layers and impairment of arterial distensibility [24]. A study has shown that higher levels of apelin cause a reduction in peripheral vascular resistance, a decrease in blood pressure [25], and an increase in the contractile reserve [26]. Shang et al. [27] found a negative correlation between the omentin concentration in circulating blood and thickness of the intima-media layer, carotid artery stiffness, and systolic blood pressure. The authors conclude that there is a close relationship between blood concentration of this adipokine and atherosclerosis. In the present study, lower levels of regulatory peptides were found in children with type 1 diabetes as compared to their healthy peers. The relationship may be important in the development of cardiovascular complications in type 1 diabetes and requires further long-term studies.

The differences detected in the study population encourage a more detailed analysis of the observed correlations and a potential contribution of these substances to the development and course of type 1 diabetes.

To conclude it should be stated that the peptides of the gastrointestinal tract and adipokines can modulate insulin sensitivity not only in healthy individuals but also in patients with pancreatic dysfunction. Their concentration not only affects the occurrence of hypo- and hyperglycemia [28], but above all, exerts long-term effects on energy homeostasis. Accordingly, the levels of the above mentioned active substances differ depending on the metabolic control, BMI, and also diabetes duration. The levels of gastric peptides and adipocytokines are higher in patients with normal renal secretion of the pancreas than in patients suffering from type 1 diabetes. In addition, the levels of ghrelin and the other substances differ between patients with preserved residual pancreatic secretory function (in remission) and those with long-lasting type 1 diabetes. Our preliminary results obtained from patients with type 1 diabetes indicate a trend in relation to the changes in the “pool” of peptides secreted in the digestive tract and adipokines released by adipocytes, which may be of importance in the development of the disease complications.

Based on data obtained from animal studies and adults with type 2 diabetes it seems that the lower concentrations of gastrointestinal peptides and adipocytokines are responsible for lower insulin sensitivity and glucose metabolism in comparison with the general population. Further research into the correlation observed should be performed on a larger group of patients, to specify the role of these substances in the course of type 1 diabetes.

## 5. Conclusions

- The levels of gastric peptides and adipocytokines are higher in patients with normal renal secretion of the pancreas than in patients with type 1 diabetes, which may be important in the development of complications in the course of this disease entity.
- The disease duration, HbA1c, and BMI can affect the levels of the regulatory peptides in the body.

## Figures and Tables

**Table 1:** The concentrations of ghrelin, obestatin, omentin-1, and apelin in blood serum of the study groups.

Hormone	Subgroup I	Subgroup II	Subgroup III	Subgroup IV	Control group	*p*, ^*∗*^ *p*, ^*∗∗*^ *p*, ^*∗∗∗*^ *p*
Ghrelin	125.2	124.6	124.8	122.7	164.5	*p* < 0.0001 ^*∗*^ *p* < 0.0001 ^*∗∗*^ *p* < 0.0001 ^*∗∗∗*^ *p* < 0.0001

Omentin-1	124.8	124.2	123.2	122.4	157.1	*p* < 0.0001 ^*∗*^ *p* < 0.0001 ^*∗∗*^ *p* < 0.0001 ^*∗∗∗*^ *p* < 0.0001

Obestatin	122.9	122.6	122.2	119	155.4	*p* < 0.0001 ^*∗*^ *p* < 0.0001 ^*∗∗*^ *p* < 0.0001 ^*∗∗∗*^ *p* < 0.0001

Apelin	124.1	122.4	123.0	120.7	151.3	*p* < 0.0001 ^*∗*^ *p* < 0.0001 ^*∗∗*^ *p* < 0.0001 ^*∗∗∗*^ *p* < 0.0001

*p*: statistical significance between subgroup I and control group; ^*∗*^ *p*: statistical significance between subgroup II and control group; ^*∗∗*^ *p*: statistical significance between subgroup III and control group; ^*∗∗∗*^ *p*: statistical significance between subgroup IV and control group.

**Table 2:** Basic characteristics of the study groups.

	Subgroup I	Subgroup II	Subgroup III	Subgroup IV	Control group
Age (years)	10.4 (*p* = 0.029)	11.1	14.3	15.5	13.4
Height (cm)	143.4	143.4	157.6	161.1	156.9
Body mass (kg)	35.9 (*p* = 0.028)	42.3	54	61.6	50.2
BMI (kg/m^2^)	18.3	18.7	21.8	23.5 (*p* = 0.027)	19.7

## References

[1] Sawicka B., Bossowski A., Szalecki M. (2010). Relationship between metabolic parameters and thyroid hormones and the level of gastric peptides in children with autoimmune thyroid diseases. *Journal of Pediatric Endocrinology and Metabolism*.

[2] Bossowski A., Czarnocka B., Harasymczuk J. (2013). Identification of GPR39 receptor and ghrelin receptor in thyroid tissues in paediatric patients with immune and non-immune thyroid diseases. *Hormone Research in Paediatrics*.

[3] Yang R.-Z., Lee M.-J., Hu H. (2006). Identification of omentin as a novel depot-specific adipokine in human adipose tissue: possible role in modulating insulin action. *American Journal of Physiology—Endocrinology and Metabolism*.

[4] Fukuhara A., Matsuda M., Nishizawa M. (2005). Visfatin: a protein secreted by visceral fat that mimics the effects of insulin. *Science*.

[5] Yoo H. J., Hwang S. Y., Hong H. C. (2011). Association of circulating omentin-1 level with arterial stiffness and carotid plaque in type 2 diabetes. *Cardiovascular Diabetology*.

[6] de Souza Batista C. M., Yang R.-Z., Lee M.-J. (2007). Omentin plasma levels and gene expression are decreased in obesity. *Diabetes*.

[7] Dray C., Knauf C., Daviaud D. (2008). Apelin stimulates glucose utilization in normal and obese insulin-resistant mice. *Cell Metabolism*.

[8] Lee D. K., Cheng R., Ngyuen T. (2000). Characterization of apelin, the ligand for the APJ receptor. *Journal of Neurochemistry*.

[9] Lazarczyk M. A., Lazarczyk M., Grzela T. (2003). Ghrelin: a recently discovered gut-brain peptide. *International Journal of Molecular Medicine*.

[10] Castañeda T. R., Tong J., Datta R., Culler M., Tschöp M. H. (2010). Ghrelin in the regulation of body weight and metabolism. *Frontiers in Neuroendocrinology*.

[11] Delzenne N., Blundell J., Brouns F. (2010). Gastrointestinal targets of appetite regulation in humans. *Obesity Reviews*.

[12] Depoortere I., Thijs T., Moechars D., De Smet B., Ver Donck L., Peeters T. L. (2008). Effect of peripheral obestatin on food intake and gastric emptying in ghrelin-knockout mice. *British Journal of Pharmacology*.

[13] Ikezaki A., Hosoda H., Ito K. (2002). Fasting plasma ghrelin levels are negatively correlated with insulin resistance and PAI-1, but not with leptin, in obese children and adolescents. *Diabetes*.

[14] Tapan S., Tascilar E., Abaci A. (2010). Decreased plasma apelin levels in pubertal obese children. *Journal of Pediatric Endocrinology and Metabolism*.

[15] Ziora K., Oświecimska J., Świetochowska E. (2010). Assessment of serum apelin levels in girls with anorexia nervosa. *Journal of Clinical Endocrinology and Metabolism*.

[16] Zamrazilová H., Hainer V., Sedláčková D. (2008). Plasma obestatin levels in normal weight, obese and anorectic women. *Physiological Research*.

[17] Reinehr T., Woelfle J., Roth C. L. (2011). Lack of association between apelin, insulin resistance, cardiovascular risk factors, and obesity in children: a longitudinal analysis. *Metabolism: Clinical and Experimental*.

[18] Duntas L. H., Orgiazzi J., Brabant G. (2011). The interface between thyroid and diabetes mellitus. *Clinical Endocrinology*.

[19] Cho Y. H., Craig M. E., Donaghue K. C. (2014). Puberty as an accelerator for diabetes complications. *Pediatric Diabetes*.

[20] Modrzejewski W., Musiał W. J. (2010). Old and new cardiovascular risk factor—how to stop an epidemic of atherosclerosis Part1. Classical risks. *Forum Zaburzeń Metabolicznych*.

[21] Kavey R.-E. W., Daniels S. R., Lauer R. M., Atkins D. L., Hayman L. L., Taubert K. (2003). American Heart Association guidelines for primary prevention of atherosclerotic cardiovascular disease beginning in childhood. *Circulation*.

[22] Giannini C., Mohn A., Chiarelli F., Kelnar C. J. H. (2011). Macrovascular angiopathy in children and adolescents with type 1 diabetes. *Diabetes/Metabolism Research and Reviews*.

[23] Broda G. (2011). Zapobiegać miażdżycy w dzieciństwie, aby zachować zdrowie w dalszych latach życia. *Kardiologia Polska*.

[24] Giannattasio C., Failla M., Piperno A. (1999). Early impairment of large artery structure and function in Type I diabetes mellitus. *Diabetologia*.

[25] Japp A. G., Cruden N. L., Barnes G. (2010). Acute cardiovascular effects of apelin in humans: potential role in patients with chronic heart failure. *Circulation*.

[26] Ashley E. A., Powers J., Chen M. (2005). The endogenous peptide apelin potently improves cardiac contractility and reduces cardiac loading in vivo. *Cardiovascular Research*.

[27] Shang F.-J., Wang J.-P., Liu X.-T. (2011). Serum omentin-1 levels are inversely associated with the presence and severity of coronary artery disease in patients with metabolic syndrome. *Biomarkers*.

[28] Malagón M. M., Luque R. M., Ruiz-Guerrero E. (2003). Intracellular signaling mechanisms mediating ghrelin-stimulated growth hormone release in somatotropes. *Endocrinology*.

